# Supplementary material for: FMRP(1–297)-tat restores ion channel and synaptic function in a model of Fragile X syndrome
Source: Nat Commun. 2020 Jun 2;11:2755. doi: 10.1038/s41467-020-16250-4 (PMC7265297; doi:10.1038/s41467-020-16250-4)
Supplement: Supplementary file 1 — Supplementary Information [file 41467_2020_16250_MOESM1_ESM.pdf]

Supplemental Table and Figures:

Zhan et al. FMRP(1-297)-*tat* restores ion channel and synaptic function in a model of Fragile X syndrome

Measure	WT	*Fmr1* KO		1.0 mg/kg FMRP(1-297)-*tat*	
	**Value**	**Value**	***p***	**Value**	***p***
**RMP (mV)**	-59.5 ± 2.3 (6)	-63.6 ± 1.8 (8)	0.18	-63.2 ± 2.8 (7)	0.92
**Input Resistance (GΩ)**	1.16 ± 0.16 (5)	0.97 ± 0.12 (6)	0.37	0.72 ± 0.05 (5)	0.12
**Firing threshold (nA)**	11.2 ± 2.0 (5)	13.2 ± 1.4 (5)	0.43	14.8 ± 1.0 (5)	0.37

Supplementary Table 1. Membrane properties of cerebellar granule cells from WT and *Fmr1* KO mice and compared to *Fmr1* KO mice prepared 2 hr after tail vein injection of 1.0 mg/kg FMRP(1-297)-*tat*. Two-Sample *t*-test was applied to compare significance between WT and *Fmr1* KO as well as WT and FMRP(1-297)-*tat* tail vein injection. Average values are mean ± s.e.m. with the number of cells recorded shown in brackets. Abbreviations: RMP, resting membrane potential. Source data are provided as a Source Data file.

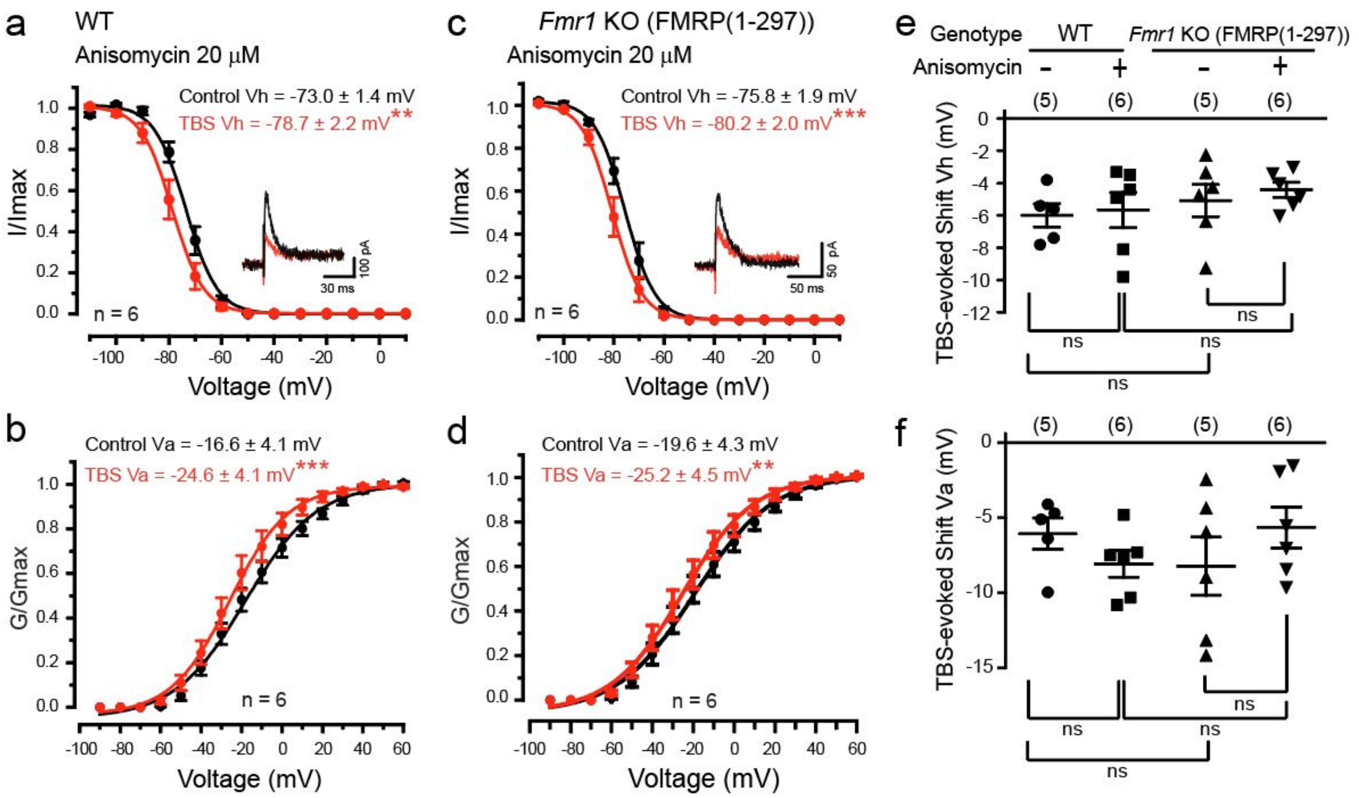

Supplementary Figure 1. The TBS-induced left shift in Kv4 Vh is independent of protein synthesis. Protein synthesis was blocked for at least 2 hr by pretreating with 20 μM anisomycin during slice recovery and in the bath medium. **a -d**, Mossy fiber TBS induces a left shift in both Kv4 Vh and Va in the presence of 20 μM anisomycin in granule cells of both WT (**a**, Paired-sample *t*-test, t(5) = 5.21, *p* = 0.003; **b**, Paired-sample *t*-test, t(5) = 8.99, *p* = 0.0003) and *Fmr1* KO mice recorded with 3 nM FMRP(1-297) in the recording electrode (**c**, Paired-sample *t*-test, t(5) = 9.382, *p* = 0.0002; **d**, Paired-sample *t*-test, t(5) = 4.12, *p* = 0.009). *Insets* in (**a, c**) show Kv4 current evoked from -70 mV to -30 mV. **e, f,** Data points and mean values for the shift in Kv4 Vh (**e**) and Va (**f**) in WT and *Fmr1* KO granule cells (with FMRP(1-297)) induced by mossy fiber TBS in tissue slices with and without anisomycin. Two way ANOVA failed to detect a significant interaction between genotype and anisomycin treatment or a difference among any two groups for the observed shifts in Va and Vh (**e**, $F_{(1,19),interaction} = 0.0361$, $p = 0.851$, $F_{(1,19),anisomycin} = 0.325$, $p = 0.575$, $F_{(1,19),genotype} = 1.526$, $p = 0.232$; **f**, $F_{(1,19),interaction} = 2.640$, $p = 0.121$, $F_{(1,19),anisomycin} = 0.038$, $p = 0.847$, $F_{(1,19),genotype} = 0.00799$, $p = 0.9297$). Average values are mean ± s.e.m. **, $p < 0.05$; ***, $p < 0.001$. Source data are provided as a Source Data file.

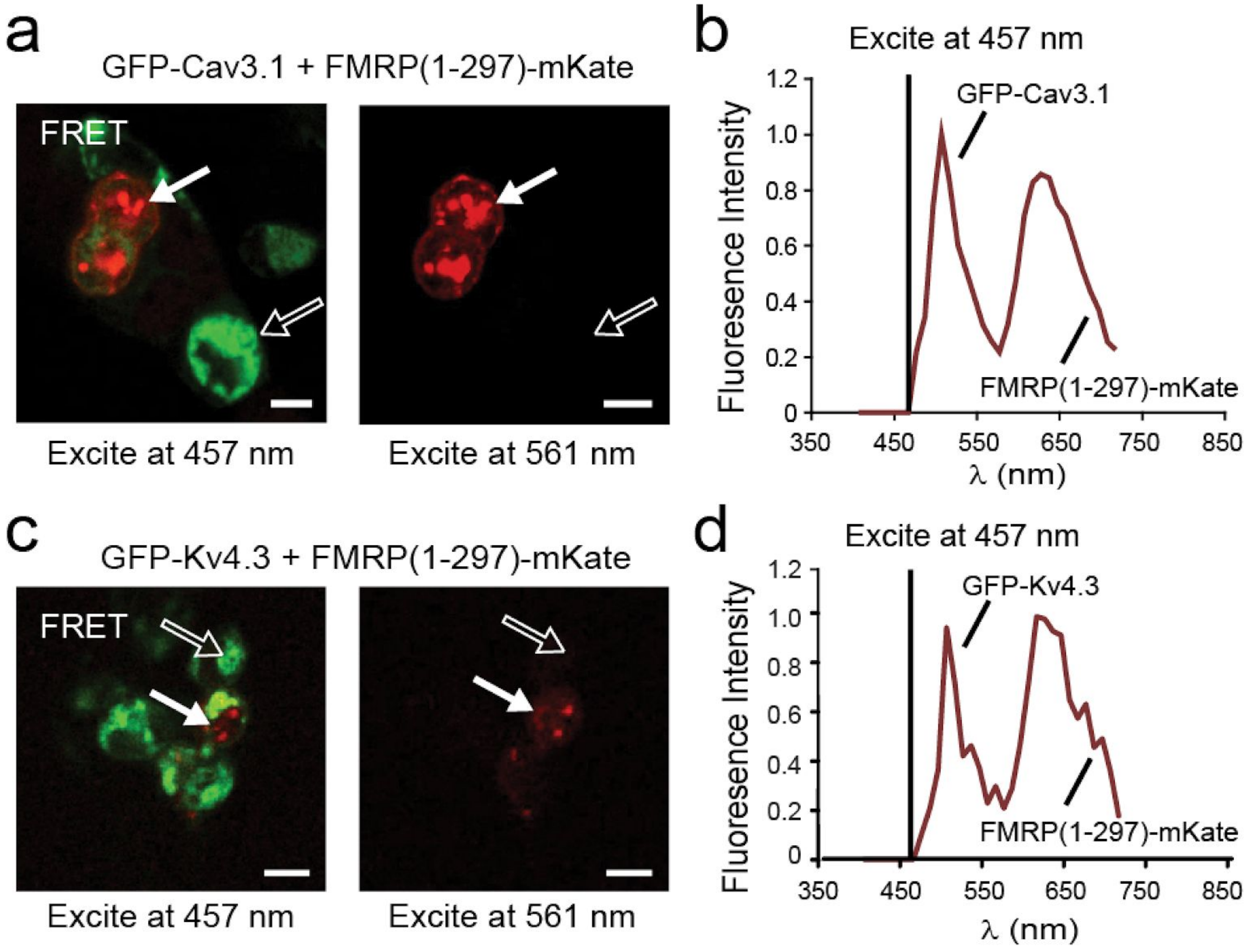

Supplementary Figure 2. Cav3.1 and Kv4.3 channels exhibit FRET with FMRP(1-297). a, b, tsA-201 cells coexpressing GFP-Cav3.1 and FMRP(1-297)-mKate as a donor-acceptor pair exhibit FRET upon excitation of GFP at 457 nm (*solid arrow*) (**a**) that is absent in cells expressing only GFP-Cav3.1 (*open arrows*). Only a red emission and no FRET is evoked upon excitation at 561 nm. A representative example of peak fluorescence emissions for both GFP and mKate indicating FRET upon excitation at 457 nm is shown in (**b**). **c, d,** tsA-201 cells coexpressing GFP-Kv4.3 and FMRP(1-297)-mKate exhibit FRET upon excitation of GFP at 457 nm (*solid arrows*) that is absent in cells expressing only GFP-Kv4.3 (*open arrows*) (**c**). No FRET is evoked upon excitation at 561 nm. A representative example of peak fluorescence emissions for both GFP and mKate indicating FRET upon excitation at 457 nm is shown in (**d**). Scale bars in (**a, c**), 10 µm. All FRET experiments were derived from 3 different experiments with at least 3 different dishes of cells. Source data are provided as a Source Data file. Software for FRET analysis can be found at: https://www.ucalgary.ca/styslab/imagetrak.

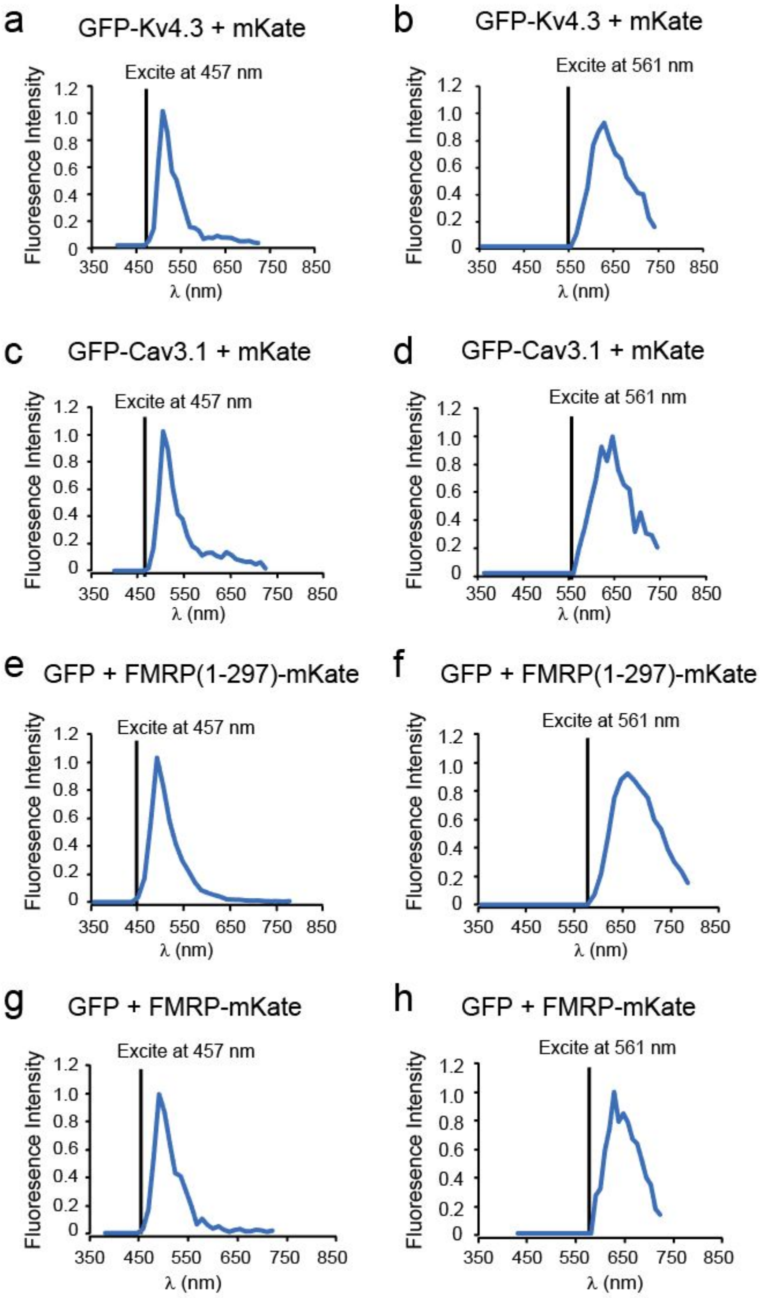

Supplementary Figure 3. FRET controls. a-h, Control tests for FRET experiments illustrated in **Figure 2** and **Supplementary Figure 2** for tsA-201 cells co-expressing the indicated cDNAs as donor-acceptor pairs. Shown are representative traces for the evoked emission profile. FRET is not indicated for any of the illustrated pairs expressed as controls, as shown by a fluorescent emission profile specific to GFP for excitation at 457 nm (**a, c, e, g**) or mKate at 561 nm (**b, d, f, h**). All control tests for FRET were derived from 3 different experiments from at least 3 different dishes of cells. Source data are provided as a Source Data file.

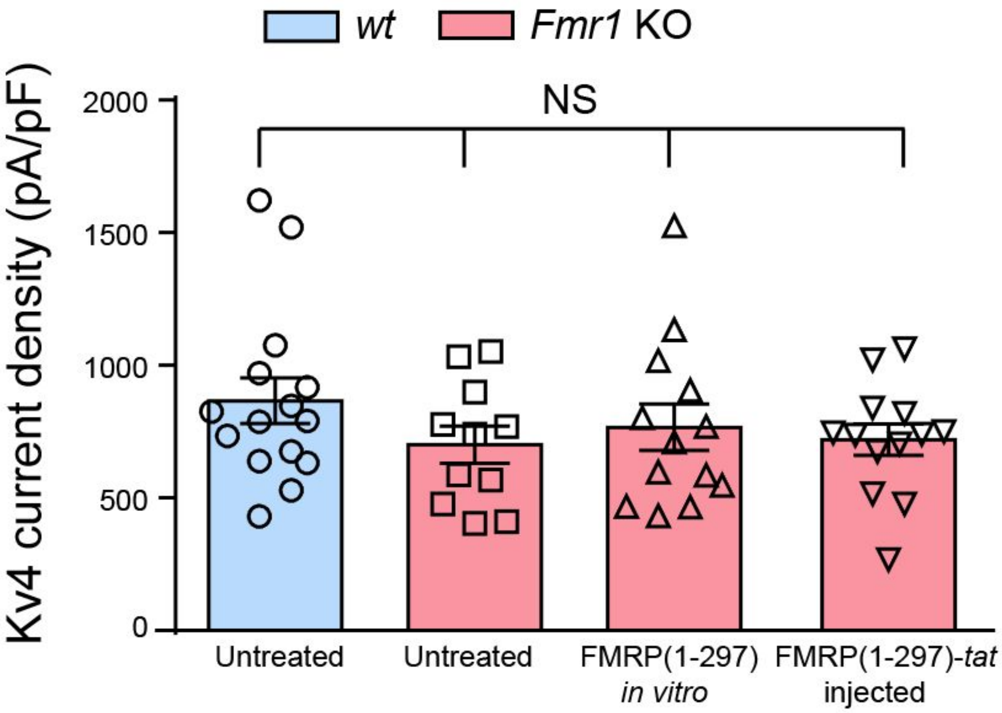

Supplementary Figure 4. FMRP(1-297) treatments do not affect maximum Kv4 current. Shown are measures of the current density for Kv4 current (pA/pF) in cerebellar granule cells recorded *in vitro* from WT (866 ± 85.5 pA/pF, n = 15 cells) or *Fmr1* KO mice (701 ± 69.89 pA/pF, n = 11 cells) to that measured after direct infusion of 30 nM FMRP(1-297) through the electrode (766 ± 87.6 pA/pF, n = 13 cells), and to recordings in slices prepared 2 hr after tail vein injection of 500 nM FMRP(1-297)-*tat* (719 ± 58.8 pA/pF, n = 13 cells). Kv4 current density was calculated for maximal Kv4 current evoked by steps from -110 mV to 60 mV and normalized with respect to measured membrane capacitance. One-way ANOVA analysis of A-type current density in these four condition cells returned no significant difference ($F_{(3,51)} = 0.94$, $P = 0.427$). Average values are mean ± s.e.m, NS, non-significant. Source data are provided as a Source Data file.

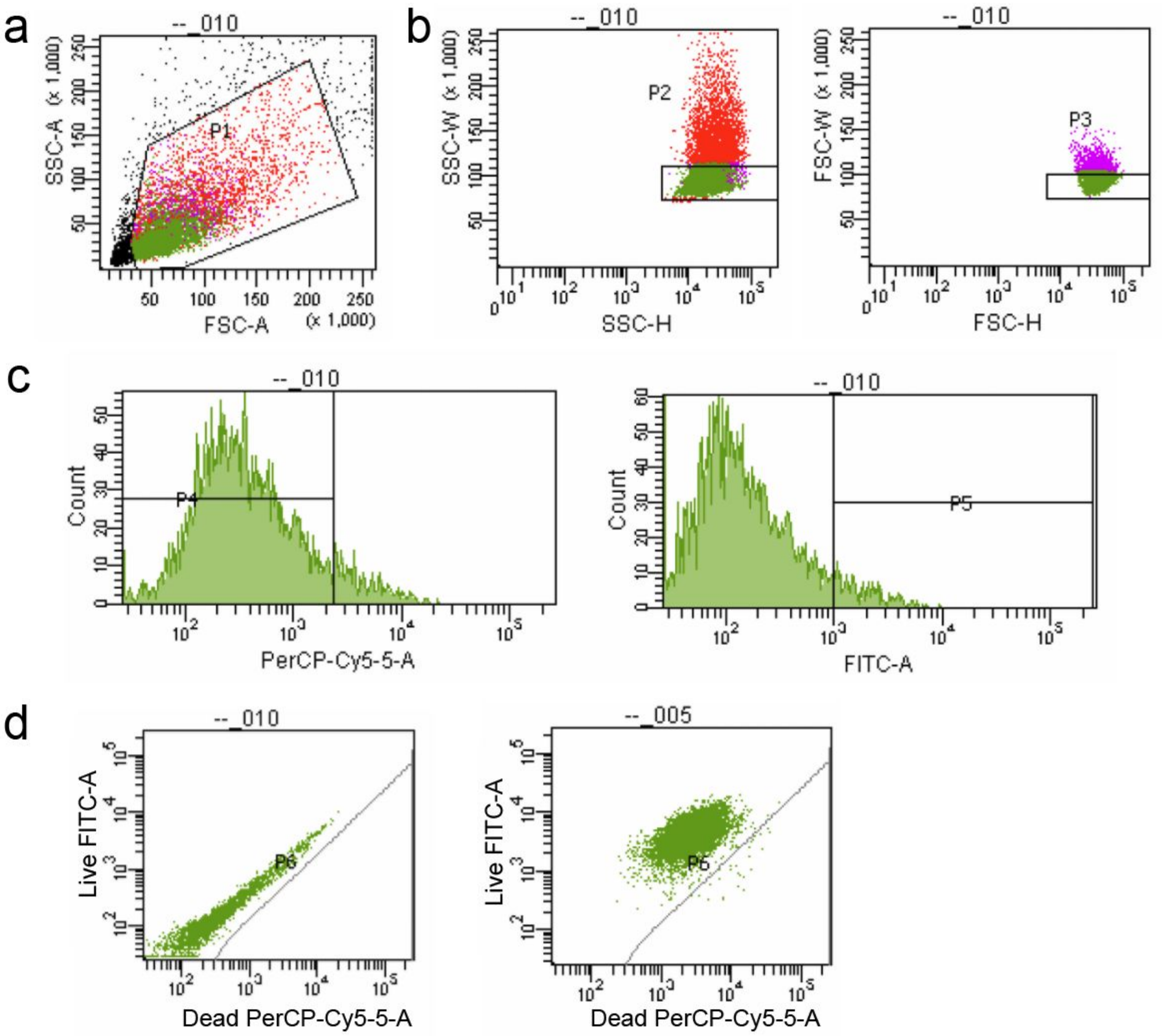

Supplementary Figure 5. Gating strategy for flow cytometric analysis of dissociated granule cells using a live-dead cell labeling kit. **a**, Plot showing the FSC/SSC gate (P1) that captures the major density of events (73.5%), eliminating 26.5% of events expected to represent debris or cell aggregations. **b**, Plots showing doublet discrimination by SSC and FSC (single-cell events retained in gates P2 and P3, respectively). **c**, Determination of positive-negative gates for the live (FITC channel – gate P4) and dead (PerCP-Cy5.5 channel – gate P5) dyes using an unstained control. **d,** Comparison between the unstained control (left, double-negative) and a stained sample (right).

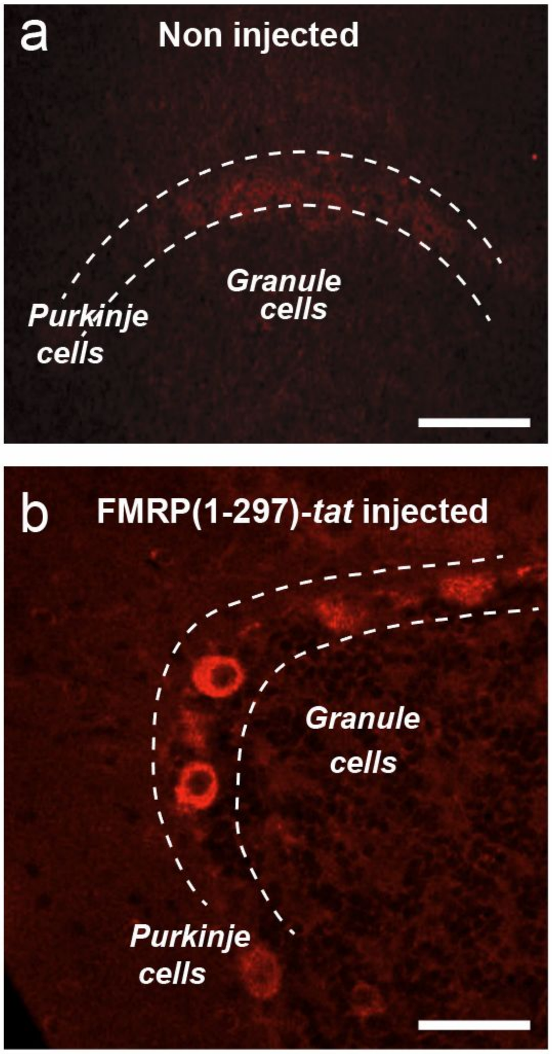

Supplementary Figure 6. FMRP(1-297)-*tat* immunolabel can be detected after 0.2 mg/kg injection. (**a, b**) Shown are representative images of immunolabel detected with an N-terminal FMRP antibody in *Fmr1* KO mouse cerebellum in a non-injected animal (**a**) or 2 hr after tail vein injection of 100 nM FMRP(1-297)-*tat* (**b**). Scale bars: 50 µm. Experiments were repeated in three animals.
