# Supplementary material for: FMRP(1–297)-tat restores ion channel and synaptic function in a model of Fragile X syndrome
Source: Nat Commun. 2020 Jun 2;11:2755. doi: 10.1038/s41467-020-16250-4 (PMC7265297; doi:10.1038/s41467-020-16250-4)
Supplement: Supplementary file 3 — Reporting Summary [file 41467_2020_16250_MOESM3_ESM.pdf]

nature research

Corresponding author(s): Turner, Ray W

Last updated by author(s): Apr 16, 2020

Reporting Summary

Nature Research wishes to improve the reproducibility of the work that we publish. This form provides structure for consistency and transparency in reporting. For further information on Nature Research policies, see Authors & Referees and the Editorial Policy Checklist .

Statistics

For all statistical analyses, confirm that the following items are present in the figure legend, table legend, main text, or Methods section.

n/a	Confirmed	
☐	☒	The exact sample size (*n*) for each experimental group/condition, given as a discrete number and unit of measurement
☐	☒	A statement on whether measurements were taken from distinct samples or whether the same sample was measured repeatedly
☐	☒	The statistical test(s) used AND whether they are one- or two-sided<br>*Only common tests should be described solely by name; describe more complex techniques in the Methods section.*
☒	☐	A description of all covariates tested
☐	☒	A description of any assumptions or corrections, such as tests of normality and adjustment for multiple comparisons
☐	☒	A full description of the statistical parameters including central tendency (e.g. means) or other basic estimates (e.g. regression coefficient) AND variation (e.g. standard deviation) or associated estimates of uncertainty (e.g. confidence intervals)
☐	☒	For null hypothesis testing, the test statistic (e.g. *F*, *t*, *r*) with confidence intervals, effect sizes, degrees of freedom and *P* value noted<br>*Give P values as exact values whenever suitable.*
☒	☐	For Bayesian analysis, information on the choice of priors and Markov chain Monte Carlo settings
☒	☐	For hierarchical and complex designs, identification of the appropriate level for tests and full reporting of outcomes
☒	☐	Estimates of effect sizes (e.g. Cohen's *d*, Pearson's *r*), indicating how they were calculated

Our web collection on statistics for biologists contains articles on many of the points above.

Software and code

Policy information about availability of computer code

Data collection	Clampex 10.3.1.4 (Molecular Devices), multiclamp 700B commander (Molecular Devices), Noldus Ethovision XT 13 (Leesburg), Image-Lab 6.0 (Bio-Rad), ZEN 2 (Zeiss)
Data analysis	pClamp 10 software (Molecular devices), Noldus Ethovision XT 13 , Origin 8 (OriginLab), GraphPad Prism 6, Image-Lab 6.0, ZEN 2 (Zeiss), Adobe Illustrator CC 2018<br><br>FRET Signal analysis: ImageTrak software used for FRET imaging measurements is freely available at: (http://www.ucalgary.ca/styslab/imagetrak; Version 4.7). P.K. Stys, University of Calgary.

For manuscripts utilizing custom algorithms or software that are central to the research but not yet described in published literature, software must be made available to editors/reviewers. We strongly encourage code deposition in a community repository (e.g. GitHub). See the Nature Research guidelines for submitting code & software for further information.

Data

Policy information about availability of data

All manuscripts must include a data availability statement. This statement should provide the following information, where applicable:

- Accession codes, unique identifiers, or web links for publicly available datasets
- A list of figures that have associated raw data
- A description of any restrictions on data availability

All relevant data are available without restriction from the authors and in the Source Date File. The original values for all data underlying Figs. 1-3, 5-9 and Supplementary Figs. 1-4 as well as Supplementary table 1 are provided as a Source Data file. Figures are freely available to public at 10.6084/m9.figshare.12132864. No accession codes are required and there are no restrictions on data availability.

Field-specific reporting

Please select the one below that is the best fit for your research. If you are not sure, read the appropriate sections before making your selection.

- [x] Life sciences
- [ ] Behavioural & social sciences
- [ ] Ecological, evolutionary & environmental sciences

For a reference copy of the document with all sections, see nature.com/documents/nr-reporting-summary-flat.pdf

Life sciences study design

All studies must disclose on these points even when the disclosure is negative.

Sample size	Sample size for in vitro electrophysiology data followed the convention of 5-10 samples from 3 or more animals or plates of tsA-201 cells. Statistical analysis was then performed to assess the statistical significance between mean values of the collected samples with two-tailed statistical tests for normally distributed data to achieve a significance of least p<0.05. Western blots were repeated with samples from at least 3 animals and FRET calculated from a minimum of 7 ROIs from 3 different experiments on tsA-201 cells. For in vivo experiments, the number of animals used is more than the number of animals calculated to satisfy a power of 0.8 and alpha equals 0.05 with our current mean values and standard deviations.
Data exclusions	Acceptance of data in current- or voltage-clamp recordings in vitro followed expected minimal standards (<20% change) for resting potential, input resistance and action potential output patterns (current-clamp), and G" seal resistance, leak current (<100 pA) ,and series resistance compensation of least 70% (voltage-clamp). Data points for behavioural work were only excluded in the case of trends that suggest a progression to adverse health or unanticipated external influences that alter the testing protocol.
Replication	Experiments were always repeated at least 3 times with different animals or samples to verify reproducibility, with some individual samples (ie coIPs, WBs) retested to further confirm a result. In all cases attempts at replication were successful.
Randomization	Animals destined for in vitro slice recordings were all male and derived from wt or Fmr1 KO animals randomly chosen for treatment according to similar age and weight. Control for covariates was not relevant to this study. Animals for protein biochemical tests were male or female and chosen primarily on the basis of close matching age and weight.
Blinding	Experimenters conducting Open Field tests for activity levels of wt, Fmr1 KO mice, or mice injected with vehicle or vehicle + HA-FMRP(1-297)-tat were blinded to the identity of mice being tested.

Reporting for specific materials, systems and methods

We require information from authors about some types of materials, experimental systems and methods used in many studies. Here, indicate whether each material, system or method listed is relevant to your study. If you are not sure if a list item applies to your research, read the appropriate section before selecting a response.

Materials & experimental systems

n/a	Involved in the study	
[ ]	[x]	Antibodies
[ ]	[x]	Eukaryotic cell lines
[x]	[ ]	Palaeontology
[ ]	[x]	Animals and other organisms
[x]	[ ]	Human research participants
[x]	[ ]	Clinical data

Methods

n/a	Involved in the study	
[x]	[ ]	ChIP-seq
[ ]	[x]	Flow cytometry
[x]	[ ]	MRI-based neuroimaging

Antibodies

Antibodies used

Primary Antibodies used
1) Rabbit polyclonal anti-Cav3.1 (Gift of Dr. G.W. Zamponi)
2) Rabbit polyclonal anti-Kv4.3 (Abcam, ab65794)
3) Rabbit IgG, monoclonal [EPR25A] - Isotope control (Abcam, ab172730)
4) Mouse monoclonal anti FMRP N-terminus [1D10] (Abcam, ab230915)
5) Rabbit monoclonal anti-FMRP C-terminal antibody (Cell Signaling Technology, 7104S)
6) Mouse monoclonal anti-HA antibody [16B12] (Abcam, ab130275)
7) Mouse IgG Isotope control (Abcam, ab37355)

8) Rabbit polyclonal anti-APP (Sigma-Aldrich, A8717)
9) Rabbit polyclonal anti- CaMKII (Abcam, ab103840)
10) Mouse monoclonal anti-PSD95 (Sigma-Aldrich, MABN68)
11) Rabbit monoclonal anti-GAPDH (Cell Signaling Technology, 2118s)
12) Mouse monoclonal anti-vinculin (Sigma-Aldrich, SAB4200729)
13) Chicken polyclonal anti-MAP2 (1:500, Abcam, ab92434)

Secondary antibodies
14) Goat anti-mouse HRP-conjugated secondary antibody (Invitrogen, 62-6520)
15) Donkey anti-rabbit HRP-conjugated secondary antibody (GE healthcare, NA-9340)
16) AlexaFluor 594 conjugated goat anti-mouse secondary antibody (Invitrogen, A11032)
17) AlexaFluor 488 conjugated goat anti-chicken secondary antibody (Invitrogen, A11039)

Validation

1) Rabbit anti-Cav3.1. In house source, G.W. Zamponi
Antibodies directed to the Cav3.1 calcium channel were produced by using the Cav3.1 I-II linker sequence (ELRKSLLPPLIIHTAATPMS), which corresponds to amino acids 1010–1027 (GenBank accession no. AF290212). The channel peptide was cross-linked to keyhole limpet hemocyanin before injection into rabbits, and the serum as polyclonal antibody was purified for use. This antibody was previously validated by our lab group by confirming selective immunolabeling and Western blot detection of Cav3.1 in HEK cells stably expressing Cav3.1, Cav3.2 or Cav3.3, but no labeling of HEK cells expressing Cav1.2, Cav1.4, Cav2.1, or Cav2.2. It has been further validated through application in immunocytochemistry (Molineux M.L., 2006) and coIP and western blot analysis on brain lysates and tsA-201 cells (Asmara H. et. al, 2017).

Molineux M.L. et al. (2006) Specific T-type calcium channel isoforms are associated with distinct burst phenotypes in deep cerebellar nuclear neurons. PNAS 103(14): 5555-5560.

Asmara H. et al. (2017) A T-type channel-calmodulin complex triggers alpha-CaMKII activation, Molecular Brain, 10:37.

2) Rabbit anti-Kv4.3 (Abcam, ab65794)
Validation provided by supplier at https://www.abcam.com/kv43kcnd3-antibody-ab65794.html, including a Data sheet providing a western blot, results of tests on species reactivity (human, predicted to react with mouse and rat), target immunogen corresponding to the human Kv4.3 N-terminus, a guarantee of its application in western blots, and references to previously published studies. These assurances were validated in the current study with application in western blot and coIP analysis from mouse brain lysates that detected a band at the appropriate molecular weight.

3) Rabbit IgG, monoclonal [EPR25A] - Isotope control (Abcam, ab172730)
Validation provided by supplier at: https://www.abcam.com/rabbit-igg-monoclonal-epr25a-isotype-control-ab172730.html including a Data sheet providing a western blot, images of immunolabeling tests, target immunogen of rabbit IgG conjugated to keyhole limpet haemocyanin, a guarantee of its application in immunocytochemistry, and 87 references to previously published studies.

4) Mouse monoclonal anti FMRP N-terminus [1D10] (Abcam, ab230915)
Validation provided by supplier at: https://www.abcam.com/fmrp-antibody-1d10-ab230915.html, including a Data sheet providing a western blot, images of immunolabeling tests, immunogen as a recombinant fragment corresponding to Human FMRP aa 36-279 with sequence provided, applications in WB and ICC/IF analyses, with references to previous published studies. These assurances were confirmed in the current study with western blot and coIP analysis from mouse brain lysates that detected a band at the appropriate molecular weight.

5) Rabbit monoclonal anti-FMRP C-terminal antibody (Cell Signaling Technology, 7104S)
Validation provided by supplier at: https://www.cellsignal.com/products/primary-antibodies/fmrp-d14f4-rabbit-mab/7104 including Data Sheet, images of western blot, and stated species reactivity of human, mouse, rat, monkey for use in ICC/IF or WB. The antibody was produced using a "synthetic peptide corresponding to residues surrounding Gly552 of human FMRP protein".

6) Mouse monoclonal anti-HA antibody [16B12] (Abcam, ab130275)
Validation provided by supplier at: https://www.abcam.com/ha-tag-antibody-16b12-ab130275.html, including images of a western blot and immunolabeling in CHO cells and endothelial cells expressing an HA-tagged protein, with recognition of the influenza Hemagglutinin epitope (YPYDVPDYA) positioned in the middle of protein sequences or the N- or C-terminus. Reactivity is species independent. Applications tested include WB and ICC/IF, as verified in 26 references and to previous published work, and repeated in the current study.

7) Mouse polyclonal IgG Isotope control (Abcam, ab37355)
Validation provided by supplier at: https://www.abcam.com/mouse-igg-isotype-control-ab37355.html#top-0 with affinity purification, a datasheet stating no known specificity, and tested applications that include IHC and ELISA.

8) Rabbit polyclonal anti-APP (Sigma-Aldrich, A8717)
Validation provided at: https://www.sigmaaldrich.com/catalog/product/sigma/a8717?lang=en&region=CA as a rabbit polyclonal anti-amyloid precursor protein (APP) using an immunogen consisting of a synthetic peptide corresponding to the C-terminal of human APP695 conjugated to keyhole limpet haemocyanin. Certified for use in WB with species reactivity of human, mouse or rat. Datasheet provides examples of ICC and WB.

9) Rabbit polyclonal anti- CaMKII (Abcam, ab103840)
Validation with datasheet provided at: https://www.abcam.com/camkii-alpha-antibody-ab103840.html, including images of ICC and Western blot, tested in mouse and rat, prepared against a synthetic peptide immunogen corresponding to human CaMKII alpha aa 400 to the C-terminus conjugated to keyhole limpet haemocyanin (exact sequence is proprietary).

10) Mouse monoclonal anti-PSD95 (Sigma-Aldrich, MABN68)
Validation and data sheet provided at: https://www.sigmaaldrich.com/catalog/product/mm/mabn68?lang=en&region=CA. NCBI accession no: NP_001356. Prepared against a "Recombinant protein corresponding to human PSD95" and certified for use in WB or ICC/IF with reactivity to rat, mouse or human, with image of ICC and Western blot provided.

11) Rabbit monoclonal anti-GAPDH (Cell Signaling Technology, 2118s)
Validation provided at: https://www.cellsignal.com/products/primary-antibodies/gapdh-14c10-rabbit-mab/2118 with data sheet for production as a rabbit monoclonal antibody directed against a "a synthetic peptide near the carboxy terminus of human GAPDH". Reactivity stated for Human, mouse or rat with image of western blot for GAPDH, supported by over 2800 published studies.

12) Mouse monoclonal anti-vinculin (Sigma-Aldrich, SAB4200729)
Validation provide at: https://www.sigmaaldrich.com/catalog/product/sigma/sab4200729?lang=en&region=CA
Clone VIN-11-5 purified from hybridoma cell culture as a mouse monoclonal produced against smooth muscle vinculin from chicken gizzard, tested for use in ICC/IF, WB, or IP and reactivity that includes mouse and human.

13) Chicken polyclonal anti-MAP2 (Abcam, ab92434)
Validation provided at: https://www.abcam.com/map2-antibody-ab92434.html
Prepared as chicken antibody against a recombinant full length protein corresponding to Cow MAP2. Recombinant bovine MAP2 protein expressed in and purified from E. Coli, suitable for ICC/IF or WB and reactivity against human or mouse protein, with images of ICC and Western blots.

14) Goat polyclonal anti-mouse IgG HRP-conjugated secondary antibody (Invitrogen, 62-6520)
Validation provided at: https://www.thermofisher.com/antibody/product/Goat-anti-Mouse-IgG-H-L-Secondary-Antibody-Polyclonal/62-6520 , including images of western blot and validated for ICC and WB.

15) Donkey anti-rabbit HRP-conjugated F(ab'2) secondary antibody (GE healthcare, NA-9340)
Validation provided at: https://www.sigmaaldrich.com/catalog/product/sigma/gena93401ml?lang=en&region=CA

16) AlexaFluor 594 conjugated goat anti-mouse secondary antibody (Invitrogen, A11032)
validation provided at: https://www.thermofisher.com/antibody/product/Goat-anti-Mouse-IgG-H-L-Highly-Cross-Adsorbed-Secondary-Antibody-Polyclonal/A-11032, including 10 immunostaining images and more than 200 references for validation.

17) AlexaFluor 488 conjugated goat anti-chicken secondary antibody (Invitrogen, A11039)
validation provided at: https://www.thermofisher.com/antibody/product/Goat-anti-Chicken-IgY-H-L-Secondary-Antibody-Polyclonal/A-11039, providing 2 immunostaining images and 45 references for validation.

Eukaryotic cell lines

Policy information about cell lines

Cell line source(s)	Human tsA201 cells were purchased from Sigma-Aldrich (9612129)
Authentication	no cell line was authenticated.
Mycoplasma contamination	Cell lines were not tested for mycoplasma contamination
Commonly misidentified lines (See ICLAC register)	No commonly misidentified cell lines were used in this study.

Animals and other organisms

Policy information about studies involving animals; ARRIVE guidelines recommended for reporting animal research

Laboratory animals	Wild type (Jackson Lab stock #004828, FVB.129P2-Pde6b+ Tyrc-ch/AntJ) and Fmr1 knockout (Jackson Lab stock #004624,FVB.129P2-Pde6b+ Tyrc-ch Fmr1tm1Cgr/J) mice were purchased from Jackson Lab. Experiments were conducted using

	age- and weight-matched wild type and knockout animals. Male mice P16-P22 were used for electrophysiological recordings, P30-P60 mice for immunostaining, P55-P100 mice for Open-Field Test, P30-P50 mice used to collect lysates for HA pull down and Co-IP tests, and P21-P30 mice were used for protein level study (Fig.9).
Wild animals	the study did not involve wild animals
Field-collected samples	the study did not involve samples collected from the field.
Ethics oversight	All procedures were approved by the University of Calgary Animal Care Committee under the guidance of certified veterinarians and guidelines of the Canadian Council on Animal Care.

Note that full information on the approval of the study protocol must also be provided in the manuscript.

Flow Cytometry

Plots

Confirm that:

- [x] The axis labels state the marker and fluorochrome used (e.g. CD4-FITC).
- [x] The axis scales are clearly visible. Include numbers along axes only for bottom left plot of group (a 'group' is an analysis of identical markers).
- [x] All plots are contour plots with outliers or pseudocolor plots.
- [x] A numerical value for number of cells or percentage (with statistics) is provided.

Methodology

Sample preparation	Primary cultures of cerebellar granule cells without any treatment, treated with vehicle, or a series of concentrations of FMRP (297)-tat were trypsinized and dissociated before cell suspension was incubated with freshly prepared Live and Dead Dyes for 10 min.
Instrument	Flow cytometer producer is BD Biosciences, model number is BD LSR II
Software	BD FACSDiva software 8.0.2 or 6.1.3
Cell population abundance	No sorting was conducted. Groups with a population higher than 5000 after gating were accepted for analysis.
Gating strategy	Forward scatter, side scatter and doublet discrimination were applied. The events with very low FSC and SSC, as well as those with high FSC and high SSC are eliminated. Non-dye control was used to define the boundaries of positive and negative populations.

- [x] Tick this box to confirm that a figure exemplifying the gating strategy is provided in the Supplementary Information.
